# Variability in the Application and Principles of Prehabilitation and Enhanced Recovery (VAPPER Survey) Among Colorectal Surgery Units: A National Multicentre Survey

**DOI:** 10.3390/nu18152540

**Published:** 2026-08-04

**Authors:** Carlos Cerdán Santacruz, Marta Carreras I Hoyos, Inmaculada Domínguez Serrano, Óscar Cano-Valderrama, María Luisa Reyes Díaz, Elena Viejo Martínez, Lara Blanco Terés, Mireia Merichal Resina, Marta Paniagua García-Señoráns, Noelia Ibáñez Cánovas, Francisco Javier Medina Fernández, Emilio Peña Ros, Inés Aldrey Cao, Leticia Pérez-Santiago, David Sánchez Relinque, Ainhoa Valle Rubio, Javier García-Septiem, Elena Martín Pérez, VAPPER Collaborative Study Group

**Affiliations:** 1 Colorectal Surgery Department, Hospital Universitario de la Princesa, Instituto de Investigación Sanitaria Princesa (IIS-IP), Universidad Autónoma de Madrid (UAM), 28006 Madrid, Spain; carloscerdansantacruz@hotmail.com (C.C.S.); elemartin2003@yahoo.es (E.M.P.); 2 Colorectal Surgery Department, Hospital Universitario Clínico San Carlos, 28040 Madrid, Spain; idserrano2@gmail.com; 3 Colorectal Surgery Department, Complejo Hospitalario Universitario de Vigo, Instituto de Investigación Sanitaria Galicia Sur, 36312 Vigo, Spain; oscarcanovalderrama@hotmail.com; 4 Colorectal Surgery Department, Hospital Universitario Virgen del Rocio, 41013 Sevilla, Spain; milu_1983@hotmail.com; 5 Colorectal Surgery Department, Hospital Universitario Infanta Leonor, 28031 Madrid, Spain; ele.viejo@gmail.com; 6 Colorectal Surgery Department, Hospital Universitario San Jorge, 22004 Huesca, Spain; blancotereslara@gmail.com; 7 Colorectal Surgery Department, Hospital Universitari d’Igualada, 08700 Barcelona, Spain; mireiamerichal@gmail.com; 8 Colorectal Surgery Department, Complejo Hospitalario Universitario de Pontevedra, 36071 Pontevedra, Spain; paniolas@gmail.com; 9 Colorectal Surgery Department, Hospital Clinico Universitario Virgen de la Arrixaca, 30120 Murcia, Spain; noelia.ibc@hotmail.es; 10 Colorectal Surgery Department, Hospital Universitario Reina Sofía de Córdoba, 14004 Córdoba, Spain; mefef.dr@gmail.com; 11 Colorectal Surgery Department, Hospital General Universitario Reina Sofía, 30003 Murcia, Spain; emilio.doctor@gmail.com; 12 Colorectal Surgery Department, Complexo Hospitalario Universitario de Ourense, 32005 Ourense, Spain; inesaldrey@hotmail.com; 13 Colorectal Surgery Department, Hospital Clínico Universitario de Valencia, 46010 Valencia, Spain; letperezsantiago@gmail.com; 14 Colorectal Surgery Department, Hospital Universitario Punta de Europa, 11207 Cádiz, Spain; srelinque@hotmail.com; 15 Colorectal Surgery Department, Hospital Universitario de Getafe, 28905 Madrid, Spain; ainhoavalle@hotmail.es (A.V.R.); jgseptiem@gmail.com (J.G.-S.)

**Keywords:** prehabilitation, colorectal surgery, nutritional assessment, ERAS, perioperative nutrition, frailty, malnutrition, colorectal cancer, GLIM and survey

## Abstract

**Background:** Prehabilitation has become an essential component of perioperative care in colorectal surgery, aiming to improve postoperative outcomes through multimodal interventions. However, its real-world implementation remains heterogeneous. This study aimed to evaluate the degree of implementation and variability of prehabilitation programs across colorectal surgery units in Spain. **Methods:** A national multicenter cross-sectional survey (VAPPER Survey) was conducted among colorectal surgery units. The questionnaire included 83 items addressing organizational structure, nutritional assessment, frailty screening, and multidisciplinary collaboration. Data were analyzed descriptively and expressed as frequencies and percentages. **Results:** A total of 104 hospitals participated. Prehabilitation programs were present in 79% (*n* = 82) of centers, while 21% (*n* = 22) reported no program. Nutritional screening was not performed in 21% (*n* = 22) of hospitals. Among those performing screening, MUST was the most frequently used tool (68%, *n* = 56), although considerable variability existed. Systematic use of GLIM criteria was reported in only 24% (*n* = 20), whereas 35% (*n* = 28) did not use GLIM and 41% (*n* = 34) applied it selectively. Frailty screening in patients ≥70 years was absent in 54% (*n* = 44) of centers, and geriatric involvement was limited and non-standardized. **Conclusions:** Prehabilitation is widely implemented in Spain but remains highly heterogeneous across all dimensions. The most relevant deficits are concentrated in systematic nutritional assessment, malnutrition diagnosis using standardized criteria, geriatric integration, and psychological support. Standardization of protocols, strengthening of multidisciplinary teams, and enhanced training in perioperative nutrition are priorities for optimizing outcomes in colorectal surgery.

## 1. Introduction

Prehabilitation has emerged over the last decade as an essential component of perioperative care in patients undergoing colorectal surgery. Defined as a preoperative multimodal intervention combining structured physical exercise, nutritional optimization, and psychological support, prehabilitation aims to improve the patient’s functional capacity before the surgical stress, with the goal of reducing postoperative complications and accelerating recovery [1,2,3].

In this context, the patient’s nutritional status plays a central role. Malnutrition is highly prevalent in patients with colorectal cancer, with reported rates ranging from 20 to 50% depending on the diagnostic criteria used and is consistently associated with increased postoperative morbidity and mortality, higher rates of surgical site infection and anastomotic dehiscence, prolonged hospital stay, and impaired quality of life [4,5]. A 2026 network meta-analysis comparing different prehabilitation modalities in colorectal cancer surgery confirmed that combined exercise, nutrition, and psychological prehabilitation produced the most consistent improvement in postoperative functional recovery, although evidence remains inconsistent regarding hospital stay, readmission, and complication rates across modalities [6]. Early identification of malnutrition through validated screening tools, followed by formal diagnosis using standardized criteria such as the GLIM (Global Leadership Initiative on Malnutrition) criteria and preoperative correction, represents a Grade A recommendation in the major international guidelines, including the European Society for Clinical Nutrition and Metabolism (ESPEN) guideline on clinical nutrition in surgery [7,8].

The ESPEN guidelines on clinical nutrition in surgery, updated in 2025, explicitly state that nutrition must be integrated into the overall management of the surgical patient from the preoperative phase onwards. Specifically, they recommend systematic nutritional screening in all candidates for major surgery, the use of GLIM criteria for malnutrition diagnosis, preoperative nutritional optimization in patients with identified risk, and the prescription of hypercaloric, high-protein oral nutritional supplements (ONSs)—or immunonutrition with arginine, omega-3 fatty acids, and nucleotides—for at least 7–14 days before surgery in high-risk patients [7,8,9]. However, despite the strength of these recommendations, their implementation in routine clinical practice remains incomplete and variable. It should be noted that the ERAS (Enhanced Recovery After Surgery) guidelines, while widely adopted, include nutritional recommendations that are at times generic and less elaborated than those of the ESPEN guidelines, which may contribute to the heterogeneity observed in perioperative nutritional practices.

In parallel, the progressive ageing of the surgical population has increased the relevance of the frailty syndrome as a major determinant of perioperative risk. Frailty is associated with worse postoperative outcomes, higher complication rates, functional decline, and increased mortality [10]. The Comprehensive Geriatric Assessment (CGA) has been proposed as the gold standard for frailty evaluation in elderly surgical patients, with demonstrated capacity to identify intervention targets and improve perioperative prognosis [11]. Nevertheless, its integration into prehabilitation pathways remains limited in most centers.

Despite growing evidence supporting prehabilitation, its real-world implementation across healthcare institutions likely varies considerably according to available resources, organizational structure, and local clinical culture. A recent systematic review of 89 studies (13,383 patients) found that participation rates in prehabilitation programs ranged from 0% to 99.4% and adherence rates from 15% to 100%, with logistical constraints, medical limitations, and psychological factors identified as the main barriers, while professional guidance and social support acted as key facilitators [12]. Understanding this variability is essential to identify key care gaps and opportunities for improvement.

The aim of the present study was to evaluate the current state of implementation of prehabilitation in colorectal surgery units across Spain, focusing specifically on perioperative nutritional practices and the integration of geriatric assessment within prehabilitation programs.

## 2. Materials and Methods

The VAPPER study was designed as a national multicenter cross-sectional survey targeting colorectal surgery units across Spain.

To select the candidate centers to be invited, we consulted the official database for the accreditation process of colorectal surgery units in our country, which falls under the remit of the Spanish Association of Coloproctology (AECP). In addition, we identified some regional leaders (AV, DS, LB, MM, OC and EP) who helped us to identify additional centers not included in this official AECP accreditation process. Finally, we reviewed the authorship of the nationally produced document on Enhanced Recovery for Adult Surgery (Recuperación intensificada en Cirugía del Adulto—RICA), identifying all the centers represented.

As there is no official national system for centralizing data on colorectal pathology, we cannot guarantee that all centers operating on patients with colon or rectal cancer in our country are represented. No official national registry of colorectal surgery units exists in Spain, and accreditation by the two national scientific societies involved in this process is voluntary; consequently, the true denominator of eligible centers nationwide cannot be established, and no claim of national representativeness is made.

A personal invitation was sent to a colorectal surgeon at 135 hospitals throughout Spain performing colorectal surgery, of which 104 responded to the survey, yielding a response rate of 77% (104/135). Data collection took place over a two-month period, from 1 June to 1 August 2025. The regional distribution of the 104 responding hospitals across Spanish Autonomous Communities is reported in Supplementary Table S1. Only one response per hospital was allowed, and no duplicate responses were generated, as each surgeon and their hospital of origin were identified during data collection. A structured questionnaire of 83 items was developed to capture detailed information across multiple perioperative care domains: institutional characteristics, prehabilitation program structure, nutritional assessment and management, frailty screening and geriatric integration, preoperative anemia management, exercise prescription, psychological support, patient education, and use of digital technologies.

The questionnaire was designed based on the clinical practice domains outlined in the ERAS guidelines for colorectal surgery and the RICA clinical pathway, together with additional items considered relevant within prehabilitation programs. Content review was carried out by two colorectal surgeons with substantial clinical and organizational experience in this field: J.G.-S and C.C.-S. Formal cognitive pretesting or external psychometric validation was not undertaken, as the questionnaire captured factual, institutional-level information on clinical practices and organizational structure rather than subjective constructs or patient-reported experiences and was administered exclusively in the respondents’ native language (Spanish). The survey was subsequently built using one of the existing free online platforms designed for this purpose (Google Forms^®^). The complete 83-item instrument, in its original Spanish language and English, is included as Supplementary Sections S2 and S3.

During the preparation of this manuscript, generative artificial intelligence tools, including Claude (Anthropic), were used to create graphical representations based on the study dataset and to assist with language editing and translation. These tools were used under the supervision of the authors, who critically reviewed, edited, and validated all generated content. The authors take full responsibility for the final version of the manuscript and its scientific accuracy.

This study consisted of an anonymous, voluntary survey assessing institutional clinical protocols and organizational practices among colorectal surgeons, involving no patients, biological samples, or clinical records. Under Spanish Royal Decree 957/2020, which governs clinical trials and observational studies involving medicinal products in human subjects, this study design falls outside the scope of research requiring Research Ethics Committee (CEIm) oversight; accordingly, ethical review and approval were waived, and no committee review or exemption reference applies. This approach is consistent with the Declaration of Helsinki’s provisions for non-interventional research not involving human subjects as research participants in the clinical sense. All participants were informed in writing of the study’s purpose, the voluntary nature of participation, and the anonymity of their responses prior to accessing the questionnaire; completion of the survey was considered implied informed consent. No sensitive personal data were collected, and data processing complied with the EU General Data Protection Regulation (GDPR).

In developing the project and this manuscript, we followed the recommendations set out in the ‘Checklist for Reporting of Survey Studies’ (CROSS) [13].

### Statistical Method

A descriptive analysis was carried out of the demographic variables and the responses to the questions, all of which were categorical in nature; therefore, the data are expressed as percentages and frequencies. Bivariate exploratory comparisons were performed on the categorical variables related to the type of unit—accredited (advanced- or basic-accreditation units) versus non-accredited (units without accreditation or with no formally constituted unit)—using the chi-square test. Cramér’s V was calculated as a measure of effect size for all comparisons, and odds ratios (ORs) with 95% confidence intervals (CIs) were additionally calculated where clinically interpretable. These comparisons were not prespecified in the original study protocol and were introduced during the review process to further characterize practice variation by accreditation status. Given the exploratory nature of these analyses and the number of comparisons performed (nine), *p*-values were not adjusted for multiple testing; a Bonferroni-corrected significance threshold would be α = 0.0056. The level of statistical significance was set at *p* < 0.05. The statistical software IBM SPSS Statistics^®^ version 24.0 (IBM, Armonk, NY, USA) was used.

## 3. Results

### 3.1. Hospital Characteristics and Prehabilitation Implementation

A total of 104 hospitals were included in the analysis, from a total of 135 invited hospitals, corresponding to a participation rate of 77%. Regarding regional distribution, 21 of the responding hospitals were located in the Community of Madrid, 15 in Catalonia, and 13 in Andalusia. Of the participating hospitals, 99 (95%) were public and 5 (5%) were private. Table 1 summarizes the baseline characteristics of participating centers. The majority were public university hospitals 88% (*n* = 92), with a tertiary care level in 50% of cases (*n* = 52). Regarding the structure of colorectal surgery units, 42% (*n* = 44) had an advanced accredited unit, while 47% (*n* = 49) had no specific accreditation.

The majority of centers, 79% (*n* = 82), reported having an established prehabilitation program, while 21% (*n* = 22) did not. A formal hospital clinical pathway for prehabilitation was reported by 68% of centers (*n* = 56). Regarding program duration, the most common model was variable duration adapted to patient profile (frail: 4 weeks; robust: 2 weeks), reported in 38% (*n* = 31) of centers.

### 3.2. Multidisciplinary Team Composition and Organization

The composition of prehabilitation teams showed marked variability across centers (Table 2). Surgeons (98%) and anesthesiologists (83%) were the most consistently represented professionals. Nurses participated in 78% of programs and were the professional group most frequently assuming program coordination after surgeons. Physiotherapists or rehabilitation physicians were integrated in 84% of teams, and clinical nutritionists or endocrinologists in 78%. By contrast, the involvement of geriatricians was limited to 27% of teams, and psychologists or psychiatrists to 21%.

Regarding program coordination, surgeons were responsible in 45% of centers, followed by nurses in 39%. A dedicated additional perioperative consultation, separate from the conventional surgical visit, was available in 59% of centers with prehabilitation programs.

### 3.3. Nutritional Assessment and Management

#### 3.3.1. Preoperative Nutritional Screening

Nutritional screening practices showed significant variability across centers (Table 3). In 21% (*n* = 22) of hospitals, no nutritional screening was performed in candidates for colorectal surgery. Among centers performing screening, MUST (Malnutrition Universal Screening Tool) was the most commonly used tool (68%), although fragmented use of multiple instruments was observed: MNA (Mini Nutritional Assessment) in 17%, NRS-2002 (Nutritional Risk Screening 2002) in 10%, and other tools such as SNAQ or PONS.

Regarding the scope of nutritional assessment, only 30% (*n* = 25) of hospitals performed a systematic evaluation of all surgical patients regardless of nutritional status. A further 62% (*n* = 51) restricted assessment to patients at moderate or high risk of malnutrition, and 7% (*n* = 6) to oncological patients.

#### 3.3.2. Malnutrition Diagnosis: Use of GLIM Criteria

The use of standardized diagnostic criteria for malnutrition was strikingly limited. Only 24% (*n* = 20) of centers reported systematic application of GLIM criteria in all surgical patients. A further 41% (*n* = 34) applied them selectively and 35% (*n* = 28) did not use them at all. These findings suggest that malnutrition may be substantially underdiagnosed, with corresponding loss of opportunities for preoperative nutritional intervention.

#### 3.3.3. Morphofunctional Assessment and Body Composition

Only 16% (*n* = 13) of the hospitals with nutritional screening systematically performed an assessment of body composition or muscle function, 29% (*n* = 24) applied them selectively and 55% (*n* = 45) did not perform it under any circumstances.

In the hospitals where it was used, bioelectrical impedance analysis (BIA) was the most common tool 54% (*n* = 20), followed by handgrip dynamometry 19% (*n* = 7), CT-based muscle analysis at the L3 level for sarcopenia estimation 19% (*n* = 7), and muscle ultrasound 8% (*n* = 3). Morphofunctional assessment—particularly the estimation of skeletal muscle mass—carries significant prognostic value in oncological colorectal surgery, and its underutilization represents a relevant care gap.

#### 3.3.4. Nutritional Intervention and Supplementation

The prescription of oral nutritional supplements showed notable heterogeneity in patients with preoperative nutritional screening. In patients without malnutrition risk, supplementation was routine (always or >75% of cases) in 43% of centers and exceptional (<25%) in 10% of them. In patients at risk of malnutrition, the most common strategy was referral to the Endocrinology/Clinical Nutrition unit (60%). Hypercaloric and high-protein oral nutritional supplementation were prescribed only to patients at nutritional risk in 43% of cases, and immunonutrient-based formulas were prescribed only to patients at nutritional risk in 34%. However, among patients at nutritional risk managed directly from the surgical outpatient clinic, immunonutrient-based oral supplements were prescribed in only 5% of cases, compared to 25% who received high-protein formulas.

#### 3.3.5. Nutritional Pathways and Protocols

Structured nutritional management pathways with clearly defined indications, follow-up protocols, and coordination with surgical planning were not universally implemented. In many centers, responsibility for nutritional management rested with the surgical team, while in others it was delegated to endocrinology or multidisciplinary teams. This variability in responsibility allocation limits continuity and standardization of perioperative nutritional care.

### 3.4. Preoperative Anemia Management

Preoperative anemia is a frequent comorbidity in patients with colorectal cancer, with well-documented impact on surgical outcomes and transfusion requirements. The results demonstrate considerable progress in anemia screening (Table 4): 87% of centers screened all pathway patients routinely. However, therapeutic management and organizational responsibility showed important variability.

Anemia treatment was managed primarily by the anesthesia service, including the conventional pre-anesthetic visit (25%) and a dedicated blood conservation clinic (19%). Intravenous iron was the most widely used therapeutic option (92%), followed by oral iron (74%). There was a specific hospital protocol for preoperative anemia management in 70% of the centers.

### 3.5. Frailty Assessment and Geriatric Involvement

#### 3.5.1. Frailty Screening

Systematic frailty screening in patients aged ≥70 years was not performed in 54% (*n* = 44) of centers (Table 5). Among those that did screen, the Clinical Frailty Scale (CFS) was the most widely used tool (29%), followed by the Fried Frailty Phenotype (24%).

The absence of exclusion criteria for prehabilitation was the norm in 76% (*n* = 62) of centers, although some reported excluding non-oncological patients 12% (*n* = 10), those with severe functional impairment (ASA IV) 6% (*n* = 5), or non-frail patients 6% (*n* = 5).

#### 3.5.2. Comprehensive Geriatric Assessment

CGA following a positive frailty screening result was not performed in 50% (*n* = 19) of centers that performed screening. Only 45% (*n* = 17) implemented it systematically. In centers where CGA was conducted, geriatric medicine was responsible in 58% of cases.

Geriatricians as permanent members of the prehabilitation team were present in only 27% of centers.

### 3.6. Exercise, Psychological Support, and Patient Education

Physical exercise was included as a component of the prehabilitation program in 91% (*n* = 75) of centers with an established program. However, there were significant differences in the type of exercise prescribed, intensity, session duration, and the professional responsible for its supervision. In many cases, prescription was managed by physiotherapists or rehabilitation physicians, although it was also handled by surgery or anesthesia teams.

Psychological support was present only in 44% (*n* = 36) of prehabilitation programs, but its implementation was variable and frequently limited to selected cases. Patient education schools or group sessions were used in approximately 27% (*n* = 22) of centers, with nutrition and rehabilitation being the most represented disciplines. Digital technologies—mobile applications or wearables—for prehabilitation delivery were rarely used routinely (6%), although willingness to adopt them was high, 99% willing to try them if evidence of effectiveness was demonstrated).

Health-related quality of life measurement using validated instruments (EQ-5D, QoR-15, SF-36, QLQ-C30) was performed in 26% (*n* = 21) of centers with prehabilitation programs, and PROMs/PROMIS were regularly used in 13% (*n* = 11).

### 3.7. Preoperative Lifestyle Optimization and Surgical Risk Assessment

Assessment of smoking habits, alcohol consumption and an active recommendation of cessation were performed in >80% of centers.

The use of surgical risk calculators in hospitals with prehabilitation program was heterogeneous: 36% (*n* = 29) of centers used them only in specific cases, 10% (*n* = 8) routinely, and 9% (*n* = 7) only for academic interest without clinical application. The most frequently used tools were P-POSSUM/CR-POSSUM and the ACS NSQIP surgical risk calculator.

### 3.8. Association Between Colorectal Surgery Unit Accreditation and Prehabilitation/Nutritional Practice

Accredited units performed systematic frailty screening in patients aged ≥70 years significantly more often than non-accredited units (60% vs. 34%; *p* = 0.017, OR = 2.96, 95% CI 1.20–7.30). No other significant differences were found between accredited and non-accredited units: the presence of an established prehabilitation program (83% vs. 76%, *p* = 0.403), the existence of a specific clinical pathway for prehabilitation (72% vs. 65%, *p* = 0.618), preoperative nutritional screening performed (83% vs. 76%, *p* = 0.403), a systematic nutritional assessment scope covering all surgical patients (37% vs. 25%, *p* = 0.259), systematic use of GLIM criteria (33% vs. 17%, *p* = 0.095), systematic morphofunctional assessment (14% vs. 17%, *p* = 0.667), frequent/routine oral nutritional supplementation in patients without malnutrition risk (59% vs. 74%, *p* = 0.136), and completion of a Comprehensive Geriatric Assessment after a positive frailty screen (48% vs. 52%, *p* = 0.746) (Table 6). Effect sizes were small to moderate across all comparisons (Cramér’s V 0.048–0.264), and the wide confidence intervals suggest limited precision given the sample size. This single nominally significant finding did not survive correction for multiple comparisons and should be interpreted as hypothesis-generating rather than confirmatory.

## 4. Discussion

This national multicenter survey provides a descriptive overview of self-reported prehabilitation practices in colorectal surgery units across Spain. Because the study captures institutional practices rather than patient-level outcomes, the findings should be interpreted as a picture of current implementation and as a set of implementation priorities, rather than as evidence that any specific practice improves postoperative outcomes in this cohort. The results show that, while prehabilitation is broadly adopted—with nearly 79% of centers reporting established programs—its real-world application is highly heterogeneous, with relevant care deficits particularly concentrated in the nutritional domain and geriatric integration.

One of the most relevant findings is the persistent deficit in perioperative nutritional care. It is important to distinguish, as separate steps, nutritional screening (a rapid, low-cost process to identify patients “at risk” who require further assessment), malnutrition diagnosis (a structured process to confirm and grade malnutrition once risk has been identified), morphofunctional assessment (quantification of muscle mass and function), and nutritional intervention (the therapeutic response once risk or diagnosis has been established). These steps are sequential and should not be conflated.

More than one in five centers (21%) performed no nutritional screening at all, and among those that did, the wide variability in tools used reflects the absence of a clear consensus on the optimal screening instrument. This situation is paradoxical in light of the ESPEN guidelines, which since 2017 have explicitly recommended systematic nutritional screening in all surgical patients [5,6,7,8].

The limited use of GLIM criteria for malnutrition diagnosis (reported systematically in only 24% of centers) is particularly striking and, given how infrequently it is used, deserves to be one of the central points of this discussion rather than a secondary observation. It is worth emphasizing that GLIM is a diagnostic framework, applied after risk has already been identified through screening—it is not itself a screening tool [14]. The GLIM criteria combine phenotypic domains (weight loss, low body mass index, reduced muscle mass) with etiologic domains (reduced food intake/assimilation, inflammation or disease burden), and were developed by consensus among the major international clinical nutrition societies to provide a common diagnostic language across settings [14]. Their scarce implementation evidences a clear translation gap between guideline recommendations and clinical practice, which may result in malnutrition underdiagnosis, inconsistent severity grading, and missed intervention opportunities. Targeted training of surgical professionals in the sequential screen–diagnose–treat pathway, and specifically in the interpretation of GLIM domains, should be considered a priority within perioperative nutrition education programs.

Regarding nutritional intervention, immunonutrient-based formulas were prescribed only to patients at nutritional risk in 34%, but only 5% of prescriptions were issued directly by surgeons. These recommendations should not be read as supporting indiscriminate or universal use of immunonutrition in all patients undergoing prehabilitation. ESPEN guidelines recommend immunomodulatory formulas—containing arginine, omega-3 fatty acids, and nucleotides—specifically in patients undergoing major surgery for gastrointestinal neoplasia who present with malnutrition or an elevated risk of complications, and always within the context of local institutional protocols [7,8]. Prescription should therefore remain linked to documented nutritional risk or diagnosed malnutrition, the oncological nature of the surgery, and locally agreed protocols, rather than being applied as a blanket recommendation.

The limited assessment of body composition constitutes another relevant care gap, and one that merits deeper discussion than a purely descriptive mention. Sarcopenia—defined by the revised EWGSOP2 consensus as a primary muscle disease characterized by low muscle strength, confirmed by reduced muscle quantity or quality, with physical performance used to grade severity (dynapenia representing isolated loss of strength without confirmed loss of mass)—is an independent predictor of postoperative complications in colorectal cancer patients [15]. Yet 55% of centers performed no muscle mass estimation. Clinically applicable tools with growing evidence in this setting include CT-based skeletal muscle index measured at the L3 vertebral level (a validated proxy for whole-body muscle mass already available in most patients undergoing routine oncological staging CT), bioelectrical impedance analysis, muscle ultrasound (increasingly used at the bedside to assess both muscle quantity and echogenicity-based quality), and handgrip dynamometry as a functional correlate of strength. Their systematic integration into prehabilitation protocols, alongside GLIM-based diagnosis, should be considered a priority objective rather than an optional refinement.

Regarding frailty, our findings are consistent with international literature highlighting the gap between recommendation and practice. Frailty itself is an established, independent predictor of worse perioperative outcomes and resource use, even outside the elective oncological setting [9,10], and Comprehensive Geriatric Assessment has demonstrated benefit in improving outcomes among older hospitalized patients more broadly [11]. In a multicenter randomized trial, multimodal (trimodal) prehabilitation was associated with improved postoperative functional recovery in patients with colorectal cancer, including a substantial proportion of older or frail patients, although the trial itself does not establish that any single component in isolation drives this benefit [16]. Our findings—in which over 54% of centers performed no frailty screening in patients aged ≥70 years and 50% did not implement a CGA after a positive screen—reveal a first-order opportunity for improvement. The integration of geriatricians as permanent prehabilitation team members, present in only 27% of centers, appears insufficient relative to the growing proportion of patients aged ≥70 years with multiple comorbidities and frailty undergoing colorectal surgery. Closer collaboration between surgical and geriatric teams, beyond occasional external consultation, represents an area with clear potential for improvement, though our cross-sectional design cannot demonstrate that such collaboration causally improves outcomes.

Interestingly, formal accreditation of the colorectal surgery unit was significantly associated with a higher rate of systematic frailty screening (*p* = 0.017), suggesting that accreditation processes may act as indicator of greater organizational maturity in this specific domain.

The low reported use of structured psychological support is one of the more striking findings of this survey and deserves closer interpretation. Psychological distress, anxiety, and depressive symptoms are common in patients awaiting cancer surgery and have themselves been associated with delayed recovery and reduced engagement with exercise and nutritional components of prehabilitation programs. The near-absence of formal psychological support across centers suggests that the “trimodal” conception of prehabilitation—exercise, nutrition, and psychological preparation [1,2]—is, in practice, being implemented as a largely bimodal model in Spain. This gap may partly reflect resource constraints (limited access to clinical psychologists within surgical pathways) rather than a lack of awareness of its relevance, and represents a specific target for future program design.

The variability in nutritional practice observed in this survey partly reflects a training deficit. Perioperative clinical nutrition requires specific competencies—screening tool selection, GLIM-based diagnosis, morphofunctional assessment, and criteria for ONS/immunonutrition prescription—that are not always systematically acquired during specialist surgical training. Practical, implementation-oriented continuing professional development programs addressing these specific competencies should be considered a cross-cutting strategy to improve the quality and consistency of perioperative nutritional care in Spain.

Digital technologies represent a potential strategy to address some of the logistical and resource barriers identified in this survey. A systematic review of digital tools supporting home-based prehabilitation prior to major surgery found encouraging feasibility and acceptability across formats including apps, wearable activity monitors, and remote coaching platforms [17], and a quasi-experimental study of an app-based complement to face-to-face respiratory physiotherapy in thoracic surgical patients showed a reduction in postoperative pulmonary complications compared with attendance-only physiotherapy [18]. However, implementation of these tools faces important barriers, including the digital divide, limited digital and health literacy—particularly relevant given the older age profile of many colorectal cancer patients—poor long-term adherence outside of trial conditions, difficulties among frail patients, data privacy concerns, and limited integration with existing clinical workflows. Participants in the present survey appeared receptive to adopting this type of solution, provided that sufficient supporting evidence becomes available; this openness represents a potential enabler for future implementation efforts in Spain.

In Spain, the recently updated Vía RICA (Recuperación Intensificada en Cirugía del Adulto) pathway—developed through a structured, GRADE-based consensus process involving multiple scientific societies—advocates standardized, time-bound preoperative optimization spanning nutrition, patient blood management, frailty assessment, and multidisciplinary coordination within ERAS-oriented clinical pathways [19]. Its core elements relevant to this discussion include universal nutritional screening with GLIM-based confirmation and 7–14 days of ONS/immunonutrition in high-risk gastrointestinal oncology patients, routine anemia screening with rapid intravenous iron access, systematic frailty screening in patients aged ≥70 years linked to CGA, and clearly defined multidisciplinary team governance.

Read against this framework, the VAPPER findings reveal specific, quantifiable discrepancies rather than a generic implementation gap: the absence of a sequential screen–diagnose–treat nutrition workflow in a substantial minority of centers (21% with no screening, only 24% using GLIM, 55% with no body-composition assessment), the limited integration of frailty screening with CGA (46% and 45% respectively, as above), and variable formalization of multidisciplinary team composition, including the low presence of geriatricians (27%) and clinical psychologists. These discrepancies suggest that the main barrier to full Vía RICA implementation in Spanish colorectal units is not the absence of a national reference framework, but rather its incomplete translation into local protocols and day-to-day practice—an interpretation directly supported by our survey data rather than an external recommendation superimposed on it.

These implementation gaps are not unique to Spain. A national survey of Dutch hospitals performing colorectal cancer surgery, using a comparable one-representative-per-center design, reported near-universal routine preoperative screening for frailty, nutritional status, and anemia (97%, 93%, and 94% of hospitals, respectively) [20], a considerably higher rate than observed in our sample and suggesting that high screening coverage is achievable at national scale. Conversely, a multinational survey of colorectal units evaluating adherence to ERAS principles found substantial variability in the uptake of individual components, with prehabilitation, anemia management, and postoperative nutrition among the elements showing the least consistent implementation despite their inclusion in ERAS Society guideline updates [16,21]. Taken together, these data suggest that the heterogeneity observed in our study reflects a broader, internationally recognized pattern of selective and uneven implementation of nutritional and geriatric components within otherwise well-established ERAS pathways [9], rather than a phenomenon specific to the Spanish healthcare system.

The present study has several limitations that must be considered when interpreting the results. As a survey-based cross-sectional study, data are self-reported by a single representative per hospital and may be subject to social desirability and response bias, potentially overestimating actual adherence to recommended practices. The questionnaire, although developed from established guideline recommendations and reviewed by two experienced colorectal surgeons, did not undergo formal cognitive pretesting or external psychometric validation prior to use; consequently, some items may have been open to differing interpretation across respondents, which cannot be fully excluded as a source of response variability. The cross-sectional design captures a single point in time and does not allow assessment of trends or changes in practice. The study does not include patient-level clinical outcome data, precluding any causal association between reported institutional practices and postoperative results; consequently, the priorities identified here should be understood as targets for standardization and future outcome-based research, not as proven determinants of improved recovery. Nevertheless, the inclusion of 104 hospitals across different care levels and institutional types, with a response rate of 77%, provides a broad national convenience sample to characterize the current state of prehabilitation in Spain, although the absence of a national registry of eligible centers precludes any formal assessment of selection or non-response bias.

Despite these methodological constraints, the data obtained remain highly informative, as they capture—for the first time in Spain—the substantial heterogeneity in prehabilitation and perioperative nutritional practice across colorectal surgery units of varying size, accreditation status, and institutional type, providing a real-world baseline against which future standardization efforts can be measured.

Future Perspectives: The marked heterogeneity identified across Spanish colorectal surgery units underscores the need for future multicenter implementation studies aimed at evaluating strategies to improve adherence to evidence-based prehabilitation pathways. Prospective research should assess whether standardized protocols incorporating systematic nutritional screening, GLIM-based malnutrition diagnosis, routine frailty assessment with comprehensive geriatric evaluation, and multidisciplinary coordination translate into improved postoperative outcomes, healthcare resource utilization, and cost-effectiveness. Furthermore, randomized or implementation-effectiveness studies comparing different organizational models of prehabilitation are warranted to identify the most efficient approaches for diverse healthcare settings. Future investigations should also explore the integration of digital health technologies, tele-prehabilitation, wearable devices, and artificial intelligence to enhance patient monitoring, adherence, and personalized perioperative care. Finally, longitudinal studies evaluating patient-reported outcomes, quality of life, functional recovery, and long-term survival will be essential to establish the clinical and economic value of comprehensive prehabilitation programs and support their widespread implementation.

## 5. Conclusions

Prehabilitation is widely implemented in colorectal surgery units across Spain, with nearly 80% of centers reporting established programs. However, its real-world application is highly heterogeneous, with the most relevant deficits concentrated in the nutritional, geriatric, and psychological dimensions: nutritional screening is not performed in more than one in five centers, malnutrition diagnosis using GLIM criteria remains infrequent, body composition assessment is not yet generalized, and frailty screening with geriatric integration shows the greatest margin for improvement.

These findings are consistent with international survey data reporting similarly incomplete implementation of nutritional and geriatric components within otherwise established ERAS pathways. Taken together, they suggest that standardization of nutritional and geriatric assessment protocols, together with reinforced multidisciplinary integration and targeted training for surgical professionals, may represent reasonable priorities to reduce the observed variability. As a descriptive, cross-sectional survey, this study characterizes current practice rather than its impact on outcomes; confirming whether such standardization translates into improved postoperative results will require dedicated outcome-based research.

## Figures and Tables

**Table 1:** Characteristics of participating hospitals and prehabilitation program components. Hospital type, level of care, unit accreditation, and prehabilitation program status are reported for the full sample (*N* = 104). Formal clinical pathway and program duration are reported among the 82 centers with an established prehabilitation program, as indicated in each row (*n*/*N*).

Variable (All Hospitals)	*n*/*N*	%
**Type of hospital**		
Public university hospital	92/104	88
Public non-university hospital	7/104	7
Private university hospital	5/104	5
**Level of care**		
Tertiary	52/104	50
Secondary	38/104	37
Community	14/104	13
**Colorectal surgery unit accreditation**		
No accreditation	49/104	47
Basic accredited unit	2/104	2
Advanced accredited unit	44/104	42
No dedicated unit	9/104	9
**Prehabilitation program (*n* = 104)**		
Yes	82/104	79
No	22/104	21
**Variable (Only Hospitals with Prehabilitation Program)**	***n*** **/*N***	**%**
**Formal clinical pathway for prehabilitation**		
Yes	56/82	68
No	26/82	32
**Prehabilitation program duration (*n* = 82)**		
2 weeks	7/82	9
3 weeks	11/82	13
4 weeks	18/82	22
Variable duration according to patient profile	15/82	18
≥4 weeks (frail)/2 weeks (robust)	31/82	38

**Table 2:** Multidisciplinary team structure and prehabilitation program organization among hospitals with an established prehabilitation program (*n* = 82). Team composition items are multiple-response; percentages are calculated over *n* = 82 unless otherwise indicated.

Professional/Organizational Aspect	*n*/*N*	%
**Team composition—professionals involved in prehabilitation. Multiple-response**		
Colorectal surgeon	80/82	98
Anesthesiologist	68/82	83
Endocrinologist/clinical nutritionist	64/82	78
Advanced practice nurse (APN)/case manager nurse	64/82	78
Physiotherapist/rehabilitation physician	69/82	84
Geriatrician	22/82	27
Psychologist/psychiatrist	17/82	21
Social worker	10/82	12
Internist/hematologist/other	12/82	15
**Program coordinator**		
Surgery	37/82	45
Nurses	32/82	39
Anesthesia	12/82	15
Internist/geriatrician	1/82	1
**Additional perioperative consultation (beyond the surgical visit)**		
Yes—dedicated additional consultation	48/82	59
Single integrated consultation	34/82	41
**Patient information materials**		
Detailed graphic material	46/82	56
Online material	3/82	4
Informal document	18/82	22
Verbal information only	15/82	18
**Digital technology use (apps, wearables)**		
Yes—routinely used	5/82	6
Available but selective use	12/82	15
Tried but abandoned	4/82	5
Not available	61/82	74

**Table 3:** Preoperative nutritional assessment and management practices in participating hospitals. Percentages for screening tools, assessment scope, GLIM criteria use, morphofunctional assessment, and oral nutritional supplementation are calculated among the 82 centers performing preoperative nutritional screening, as indicated in each row (*n*/*N*).

Nutritional Practice	*n*/*N*	%
**Preoperative nutritional screening**		
Yes—screening performed	82/104	79
No screening performed	22/104	21
**Screening tool used (among centers performing screening, *n* = 82) Multiple-response**		
MUST (Malnutrition Universal Screening Tool)	56/82	68
MNA (Mini Nutritional Assessment)	14/82	17
NRS-2002 (Nutritional Risk Screening 2002)	8/82	10
SNAQ/PONS/other tools/combination of previous	16/82	20
**Scope of nutritional assessment, *n* = 82**		
All surgical patients—systematically	25/82	30
Only moderate/high malnutrition risk patients	51/82	62
Only oncological patients	6/82	7
**Use of GLIM criteria for malnutrition diagnosis, *n* = 82**		
Yes—systematically in all surgical patients	20/82	24
Yes—selectively	34/82	41
No—GLIM criteria not used	28/82	35
**Morphofunctional/body composition assessment, *n* = 82**		
Yes—systematically in all surgical patients	13/82	16
Yes—selectively	24/82	29
No	45/82	55
**Oral nutritional supplementation in patients WITHOUT malnutrition, *n* = 82**		
Always	21/82	26
Routine (>75% of cases)	14/82	17
Frequent (50–75% of cases)	20/82	24
Occasional (25–50% of cases)	19/82	23
Exceptional (<25% of cases)	8/82	10

**Table 4:** Preoperative anemia screening and management practices in participating hospitals. Percentages are calculated using the denominator applicable to each item, as indicated in each row (*n*/*N*).

Variable	*n*/*N*	%
**Routine anemia screening**		
Always	90/104	87
Only oncological patients	7/104	7
Only severe anemia or anemia as primary symptom	5/104	4
Not performed—insufficient time in perioperative pathway	2/104	2
**Anemia management responsibility**		
Hematology service	23/104	22
Dedicated blood conservation clinic	20/104	19
Conventional pre-anesthetic assessment visit	26/104	25
Surgery team	24/104	23
Unknown	11/104	11
**Therapeutic options routinely used (multiple response)**		
Intravenous iron	96/104	92
Oral iron	77/104	74
Erythropoietin/erythropoiesis-stimulating agents	46/104	44
**Formal hospital protocol for preoperative anemia**		
Yes—specific protocol available	70/104	67
No protocol exists	25/104	24
Unknown	9/104	9
**Restrictive transfusion policy**		
Yes—applies restrictive criteria	80/104	77
No restrictive criteria applied	24/104	23

**Table 5:** Frailty screening and geriatric integration in hospitals with an established prehabilitation program (*n* = 82). Percentages for frailty screening tool use and Comprehensive Geriatric Assessment (CGA) are calculated among the 38 centers performing systematic frailty screening; the professional responsible for CGA is reported among centers performing CGA, as indicated in each row (*n*/*N*).

Variable	*n*/*N*	%
**Systematic frailty screening in patients aged ≥70 years**		
Yes—systematically in all elderly patients	38/82	46
No frailty screening performed	44/82	54
**Frailty screening tool used (among centres performing screening), *n* = 38**		
Clinical Frailty Scale (CFS)	11/38	29
Fried Frailty Phenotype	9/38	24
G8/Edmonton Frail Scale/combined MUST	5/38	13
Short Physical Performance Battery (SPPB)	2/38	5
Timed Up and Go/Barthel/other tools	11/38	29
**Comprehensive Geriatric Assessment (CGA) after positive frailty screening, *n* = 38**		
Yes—systematically	17/38	45
Selectively	2/38	5
No CGA performed	19/38	50
**Professional responsible for CGA, *n* = 38**		
Geriatric medicine	22/38	58
Anesthesia/perioperative medicine	8/38	21
Specially trained nursing	8/38	21
**Integration of geriatricians in the prehabilitation team, *n* = 82**		
Regular team member	22/82	27
No geriatric involvement	60/82	73

**Table 6:** Association between colorectal surgery unit accreditation status and prehabilitation/nutritional practice variables (bivariate exploratory comparisons; χ^2^ test, Cramér’s V, and odds ratios with 95% CI).

Category	Accredited Unit *n* (%)	Non-Accredited Unit *n* (%)	*p*-Value *	Cramér’s V	OR (95% CI) †
**Established prehabilitation program (*N* = 104)**					
No	8/46 (17%)	14/58 (24%)	0.403	0.082	1.51 (0.57–3.99)
Yes	38/46 (83%)	44/58 (76%)			
**Specific clinical pathway for prehabilitation (*N* = 82)**					
No	11/38 (29%)	15/44 (34%)	0.618	0.055	1.27 (0.50–3.24)
Yes	27/38 (71%)	29/44 (66%)			
**Preoperative nutritional screening performed (*N* = 104)**					
No	8/46 (17%)	14/58 (24%)	0.403	0.082	1.51 (0.57–3.99)
Yes	38/46 (83%)	44/58 (76%)			
**Systematic nutritional assessment scope (*N* = 82)**					
Not systematic	22/35 (63%)	35/47 (74%)	0.259	0.125	1.72 (0.67–4.45)
Systematic (all patients)	13/35 (37%)	12/47 (26%)			
**Systematic use of GLIM criteria (*N* = 82)**					
Not systematic	24/36 (67%)	38/46 (83%)	0.095	0.184	2.38 (0.85–6.65)
Systematic use	12/36 (33%)	8/46 (17%)			
**Systematic morphofunctional assessment (*N* = 82)**					
Not systematic	31/36 (86%)	38/46 (83%)	0.667	0.048	0.77 (0.23–2.58)
Systematic use	5/36 (14%)	8/46 (17%)			
**Frequent/routine ONS in patients WITHOUT malnutrition risk (*N* = 82)**					
Frequent/routine (≥50%)	21/36 (58%)	34/46 (74%)	0.136	0.165	2.02 (0.80–5.15)
Infrequent (<50%)	15/36 (42%)	12/46 (26%)			
**Systematic frailty screening (patients ≥ 70 y) (*N* = 82)**					
No	15/38 (39%)	29/44 (66%)	0.017	0.264	2.96 (1.20–7.30)
Yes	23/38 (61%)	15/44 (34%)			
**Comprehensive Geriatric Assessment after positive screening (*N* = 38)**					
No	10/19 (53%)	9/19 (47%)	0.746	0.053	0.81 (0.23–2.90)
Yes	9/19 (47%)	10/19 (53%)			

The denominator for each variable reflects the number of valid responses for that item among accredited (*n* = 46) and non-accredited (*n* = 58) units; variables subject to questionnaire skip logic have a reduced denominator, as indicated in each row (*n*/*N*). * *p*-value from the χ^2^ test, accredited vs. non-accredited units; *p* < 0.05 considered nominally significant. No expected cell count was <5, so Fisher’s exact test was not required. Not adjusted for multiple comparisons (Bonferroni-adjusted α for 9 comparisons = 0.0056). † OR (95% CI) compares the “Yes/Systematic/≥50%” category, accredited vs. non-accredited units. Cramér’s V effect sizes were small to moderate overall (range 0.048–0.264). The single nominally significant association (systematic frailty screening, *p* = 0.017) did not survive Bonferroni correction and should be regarded as hypothesis-generating rather than confirmatory.

## Data Availability

Given the nature of this study, which does not collect patient data but rather the results of a survey of healthcare professionals, we consider its publication in a public data repository unnecessary. In any case, the corresponding author agrees to provide the database containing the results upon well-reasoned request from other researchers.

## Supplementary Materials

The following supporting information can be downloaded at https://www.mdpi.com/article/10.3390/nu18152540/s1, Table S1: Regional distribution of responding hospitals across Spanish Autonomous Communities; Section S1: VAPPER Collaborative Study Group; Section S2: VAPPER Survey questionnaire—original Spanish version (83 items) and Section S3: VAPPER Survey questionnaire—English version (83 items).
