# Supplementary material for: Variability in the Application and Principles of Prehabilitation and Enhanced Recovery (VAPPER Survey) Among Colorectal Surgery Units: A National Multicentre Survey
Source: Nutrients. 2026 Aug 4;18(15):2540. doi: 10.3390/nu18152540 (PMC13468448; doi:10.3390/nu18152540)
Supplement: Supplementary file 1 [file nutrients-18-02540-s001.zip › nutrients-4423030-supplementary.pdf]

Supplementary Materials

Supplementary Table S1. Regional distribution of responding hospitals across Spanish Autonomous Communities.

Autonomous Community (Spain)	Responding hospitals, n	% of total sample (N=104)
Madrid	21	20.2%
Cataluña	15	14.4%
Andalucía	13	12.5%
Comunidad Valenciana	9	8.7%
Galicia	6	5.8%
Castilla y León	6	5.8%
Murcia	5	4.8%
Castilla-La Mancha	5	4.8%
País Vasco	4	3.8%
Aragón	4	3.8%
Canarias	4	3.8%
Asturias	4	3.8%
Extremadura	2	1.9%
Navarra	2	1.9%
Islas Baleares	2	1.9%
Cantabria	1	1.0%
La Rioja	1	1.0%
**Total**	**104**	**100%**

Note: This table reports the distribution of the 104 hospitals that responded to the VAPPER Survey. A parallel breakdown of all 135 invited hospitals by region, and of regional response rates, is not available, as invitations were issued through personal contacts and regional leads rather than a centralized regional log; this is acknowledged as a limitation in the main manuscript.

Supplementary Materials Section S1. VAPPER Collaborative Study Group.

Carlos Cerdán Santacruz, Marta Carreras i Hoyos, Inmaculada Domínguez Serrano, Óscar Cano Valderrama, M. Luisa Reyes Díaz, Elena Viejo Martínez, Lara Blanco Terés, Mireia Merichal Resina, Marta Paniagua García-Señoráns, Noelia Ibáñez Cánovas, Francisco Javier Medina Fernández, Emilio Peña Ros, Inés Aldrey Cao, Leticia Pérez-Santiago, David Sánchez Relinque, Ainhoa Valle Rubio, Alberto Damiano Cassiano, Marta Fernández-Nespral, Daniel Sánchez Relinque, Juan Ocaña Jimenez, Alfredo Vivas López, Carolina González Gómez, Marta Pérez González, Isabel María Gallarin Salamanca, Beatriz Arencibia-Pérez, Jorge Arredondo Chavez, Purificación Calero Garcia, Norman Jael Cervantes Juárez, José Antonio Romero González, Belen Manso Abajo, Teresa Calderón Duque, Maria Del Pilar Concejo Cutoli, Romina Pena López, Luis Miguel Jiménez, Marlén Alvite Canosa, Carlos Álvarez Laso, Daniel Fernandez Martínez, Lidia Cristobal Poch, Ana Huidobro Louredo, Vicente Simó Fernández, Maria Jose Gomez Jurado, Fernando Labarga Rodríguez, María Nagore Arana De La Torre, David Ambrona Zafra, Victoria Maderuelo Garcia, Ane Etxart Lopetegi, Natalia Alonso Hernández, Javier Rivera Castellano, Lorena Rodríguez Gómez, María Ojeda Martínez, Lourdes Avelino González, Patricia Saez Carlin, Carlos Pastor Idoate, Rafael García Domínguez, Silvia Salvans Ruiz, Guillermo Carreño Villareal, Almudena Moreno Serrano, Brenda García Argüelles, Manuela Elia Guedea, Zahira Gomez Carmona, Juan Manuel Romero-Marcos, Eduardo Alvarez Sarrado, Graciela Valero Navarro, Gianluca Pellino, María Jesús Ladra González, Zutoia Balciscueta Coltell, Manuel Romero Simó, Marina Alarcón Iranzo, José Antonio Rueda Orgaz, Elena González Sánchez-Migallón, Juan Cintas Catena, Eugenio Licardie Bolaños, David Sánchez Relinque, Irene Esquíroz Lizaur, Cristina López González, Miguel León Arellano, Carmen Jiménez Ceinos, Laura Sobrerroca Porras, Andrea Jiménez Salido, Marta Cuadrado Velazquez, Francesc Feliu Villaró, Olga Blasco Delgado,

Ventura Amador Barrameda, Raquel A Latorre Fragua, Antonella Peruzzi, Daniel Huerga Álvarez, Tatiana Gómez Sánchez, Federico Grasa González, María Del Coral De La Vega Olías, María De Los Ángeles García Martínez, Ana Soto Sánchez, Lucas Ojea Ruiz-Yherla, Paola Lora Cumplido, Antonio Álvarez García, Santiago Mera Velasco, Miguel Martínez Vilalta, Manuel Cendrero Martin, Anna Serracant Barrera, Maria Teresa Torres Sánchez, Carlos Gracia Roche, Carlos Javier Gómez Díaz, Jorge Garcia Egea, Erika Romero Pujana, Marta Hidalgo Pujol, Marta Artaza Irigaray, Lucas Casalduero García, Cristina Viyuela García, Javier García-Septiem

Supplementary Materials Section S2. VAPPER Survey questionnaire — original Spanish version (83 items).

Identificación y consentimiento

1. Nombre y Apellido *(open text)*

2. Correo electrónico de contacto *(open text)*

3. Nombre del hospital *(open text)*

Características del centro

4. Tipo de hospital
Público no universitario / Público universitario / Privado no universitario / Privado universitario

5. Nivel asistencial hospitalario
Terciario / Segundo nivel / Comarcal

6. ¿Se trata de una unidad acreditada de cirugía colorrectal?
Unidad básica / Unidad avanzada / Unidad sin acreditación / No hay unidades

7. ¿Cuál es la posición que ocupa en su servicio?
Residente / Fellow en coloproctología / Adjunto con menos de 10 años de dedicación a coloproctología / Adjunto con más de 10 años de dedicación a coloproctología / Jefe de Unidad / Jefe de Servicio

Programa de prehabilitación — Estructura general

8. ¿Existe un programa de prehabilitación preoperatoria establecido en su hospital?
Sí / No (salta a la pregunta 37)

9. En caso afirmativo, ¿existe una vía clínica específica hospitalaria para su conocimiento por parte de cualquier profesional del hospital y su correcto desarrollo?
Sí / No

10. ¿Cuál de los siguientes se encuentran en el equipo habitual de prehabilitación? *(multiple choice)*
Cirujano / Anestesista / Endocrino/nutricionista / Enfermería de práctica avanzada / Asistente social / Fisioterapeutas / Médicos rehabilitadores / Geriatras / Psiquiatras/psicólogos / Otro (respuestas observadas: Farmacia, Hematología, Internista, Neumólogo, Intensivistas, Cardiólogo)

Población diana

11. De entre los participantes del grupo, ¿quién coordina las actuaciones de los pacientes durante el periodo en que hacen la prehabilitación?
Enfermera gestora de casos / Enfermera de práctica avanzada / Anestesia / Cirugía / Geriatría / Otro (respuesta observada: Internista)

12. ¿Existe algún criterio de exclusión para la aplicación de programas tipo prehabilitación en su centro? *(multiple choice)*
Pacientes obesos / Pacientes diabéticos / Pacientes ASA IV / Pacientes jóvenes (<70 años) / Pacientes no frágiles / Pacientes metastásicos / Pacientes no oncológicos / No consideramos que haya ningún criterio de exclusión

13. ¿Entre los pacientes ancianos (mayores de 70 años) se realiza de forma sistemática un cribado de fragilidad para seleccionar a los candidatos para prehabilitación?
No / G8 / Escala de fragilidad de Fried / Test de Timed Up and Go / Edmonton Frail Scale / Short Physical Performance Battery / Clinical Frailty Scale (CFS) / Otro (respuestas observadas incluyen: Barthel, MUST, Charlson, MADiT/IF, valoración por oncogeriatría, entre otras)

14. En caso de cribado positivo, ¿se realiza una consulta para diagnóstico preciso de fragilidad mediante Valoración Geriátrica Integral (VGI)?

No / Sí / De forma selectiva

15. Si la respuesta es de forma selectiva, especifique en qué casos se realiza. *(open text)*

16. ¿Quién presta atención en esa consulta de diagnóstico de fragilidad?
Geriatría / Anestesia / Enfermería con formación dedicada a este fin / Lo desconozco / Otro

Educación preoperatoria

17. En cuanto a la información que se da al paciente en la consulta preoperatoria, ¿existe alguna consulta adicional dentro del circuito que permita específicamente hablar de toda la parte de cuidados perioperatorios, incluyendo prehabilitación y recuperación intensificada, o tiene que hacerse todo en una única consulta?
Consulta única / Existe una consulta adicional separada de la consulta quirúrgica / NS/NC

18. En caso de existir una consulta adicional como recurso para informar de todos estos aspectos, ¿quién coordina dicha consulta?
Anestesia / Enfermería de práctica avanzada / Escuela de pacientes / Geriatría / Otro (respuesta observada: Cirugía)

19. ¿Han elaborado en su centro algún tipo de documento o infografía con la que apoyar la información que dan a los pacientes de manera preoperatoria?
No se ha elaborado nada en nuestro centro; sólo información verbal / Existe un documento informal / Se ha elaborado un material gráfico detallado / Se ha elaborado un material online

Optimización preoperatoria

20. ¿Se aplican calculadoras de riesgo quirúrgico para establecer la morbi-mortalidad esperada de cada caso concreto?
No, nunca usamos nada más allá de la clasificación ASA de los anestesistas / Sólo en casos concretos de apariencia frágil / Sólo en casos concretos con diagnóstico de fragilidad / Solo se emplean de forma puntual, sin que existan criterios estandarizados en cuanto al perfil de pacientes o tipo de intervención / Los aplicamos con interés científico y académico, pero no clínico / Siempre

21. En caso de respuesta afirmativa previa, por favor señale cuál de las siguientes herramientas emplea: *(multiple choice)*
P-Possum / CR-Possum / ACS NSQIP (calculadora disponible en riskcalculator.facs.org) / E-PASS / DGAV / Índice de Riesgo Cardíaco Corregido (Índice de Lee) / Otro

22. ¿Se realiza una valoración de hábito tabáquico y, de existir, se realizan recomendaciones específicas para su abandono?
Se recoge como antecedente médico / Se recomienda el abandono / Existe infografía dedicada a esta cuestión para facilitar el abandono tabáquico / Se envía a una unidad especializada / No forma parte de la rutina habitual la valoración específicamente sobre el hábito tabáquico

23. ¿Se realiza una valoración de hábito enólico y, de existir, se realizan recomendaciones específicas para su abandono?
Se recoge como antecedente médico, nada más / Se recomienda activamente el abandono / Existe infografía dedicada a esta cuestión para facilitar el abandono enólico / Se envía a una unidad especializada / No forma parte de la rutina habitual la valoración específicamente sobre el hábito enólico

Sesiones divulgativas

24. ¿Se han realizado en los últimos 5 años sesiones divulgativas para dar a conocer los objetivos y resultados potenciales de estos programas?
Sí / No

25. En caso afirmativo, seleccione todas las opciones que correspondan acerca del público a quién iban dirigidas dichas sesiones divulgativas: *(multiple choice)*
Pacientes / Personal sanitario: enfermería y técnicos en cuidados auxiliares / Personal sanitario: fisioterapeutas / Personal sanitario: facultativos del servicio de cirugía / Personal sanitario: facultativos de otras especialidades implicadas no quirúrgicas (p. ej. anestesia, rehabilitación, endocrinología) / Personal sanitario: atención primaria / Dirección o gerencia hospitalaria

Duración

26. ¿Cuál es la duración del programa de prehabilitación?
Dos semanas / Tres semanas / Cuatro semanas / Un mínimo de cuatro semanas, y dependiendo del paciente a veces se puede prolongar / Dependiendo del perfil del paciente: pacientes frágiles cuatro semanas y pacientes no frágiles dos semanas

Enfermería de práctica avanzada (EPA)

27. ¿Cuentan con enfermería de práctica avanzada (EPA) para la atención continuada de los pacientes que se incluyen en un programa de prehabilitación?

Sí / No

28. En caso de que disponga de EPA para su circuito de prehabilitación, señale la opción que más se ajusta a su realidad: *(multiple choice)*

Realiza su propia valoración preoperatoria / Hace un seguimiento presencial de la evolución del proceso / Hace un seguimiento telefónico de la evolución del proceso preoperatorio de los pacientes / Hace un seguimiento evolutivo tras el alta hospitalaria / Sus labores son puramente administrativas; es más bien una gestora de casos que EPA

Calidad de vida (CdV)

29. ¿Utilizan alguna escala para valoración de calidad de vida de forma perioperatoria?

No / Sí / Solo en aquellos pacientes en prehabilitación candidatos a un estudio que lo requiera / NS/NC

30. En caso afirmativo, ¿qué escala se emplea? *(open text)*

PROMs y PROMIS

31. ¿Utilizan alguna escala para evaluación de los PROMs o PROMIs?

No / Sí / Solo en aquellos pacientes en prehabilitación candidatos a un estudio que lo requiera / NS/NC

32. En caso afirmativo, ¿qué escala se emplea? *(open text)*

Escuela de pacientes / sesiones grupales

33. ¿Son las escuelas para pacientes o las reuniones de grupos de pacientes concretos una herramienta habitual de uso en su centro?

Sí / No

34. En caso de que su respuesta haya sido afirmativa, señale todas aquellas disciplinas que tengan elaborados talleres o actividades de este tipo: *(multiple choice)*

Anestesia / Nutrición / Psiquiatría/Psicología / Circuito genérico perioperatorio tipo ERAS / Rehabilitación / Geriatría / Ninguna / Lo desconozco

Uso de nuevas tecnologías

35. ¿Se apoyan de alguna manera dentro de su unidad en nuevas tecnologías como herramientas digitales, aplicaciones móviles o dispositivos tipo wearables para la aplicación de principios de prehabilitación en sus pacientes?

Sí, lo recomendamos en todos los casos / Disponemos de herramientas que usamos en casos seleccionados / Lo hemos probado, pero lo hemos abandonado / No disponemos y nunca hemos probado

36. En caso de que nunca hayan trabajado en esta línea, ¿estarían dispuestos a probarlo si existiera alguna plataforma que hubiera demostrado ser satisfactoria para los pacientes?

Sí, estaríamos dispuestos a probarlo / No creo que funcione para nuestros pacientes / No creo que existan tales herramientas

Manejo de la anemia preoperatoria

37. El cribado de la anemia preoperatoria es una práctica habitual en los pacientes que entran dentro de la vía clínica de recuperación intensificada:

Siempre / Sólo los pacientes oncológicos / No hay tiempo suficiente entre la valoración preanestésica y la programación de la intervención, por lo que no se realiza este cribado / No forma parte de la rutina habitual, solo en casos con anemias graves o cuyo "síntoma" principal es la anemia

38. El manejo de la anemia, en los casos en que se diagnostica, se realiza:

Dentro de la consulta preanestésica convencional / Existe una consulta específica de "ahorro de sangre" para el tratamiento de la anemia preoperatoria / Por parte del servicio de Hematología / Por parte del servicio de Medicina Interna / El propio equipo de Cirugía maneja la anemia preoperatoria / Otro (respuestas observadas: Digestivo, Hospital de Día, entre Cirugía y Hematología)

39. ¿Cuál de las siguientes alternativas terapéuticas forman parte de su arsenal habitual en el contexto del tratamiento de una anemia preoperatoria? *(multiple choice)*

Hierro oral / Hierro intravenoso / Administración de eritropoyetina / Administración de otros fármacos estimulantes de la eritropoyesis / NS/NC

40. ¿Considera usted que su unidad aplica realmente medidas restrictivas para transfusión de concentrados de hematíes?

Sí / No / No tengo opinión al respecto

41. ¿Existe en su hospital un protocolo concreto de manejo del paciente con anemia preoperatoria en el que se establecen con claridad las distintas indicaciones de los tratamientos, controles posteriores y demás?

Sí / No / Lo desconozco

Nutrición

42. ¿Se realiza durante la valoración nutricional un pesado y tallado del paciente?

Siempre / Nunca / En función de las respuestas a las preguntas acerca de la pérdida de peso / NS/NC

43. ¿Hacen algún tipo de cribado de desnutrición para los pacientes que incluyen en el programa de prehabilitación? *(multiple choice)*

No / Mini Nutritional Assessment (MNA) / Malnutrition Universal Screening Tool (MUST) / Subjective Global Assessment (SGA) / Nutritional Risk Screening 2002 (NRS 2002) / Simplified Nutritional Assessment Questionnaire (SNAQ) / Perioperative Nutrition Screen (PONS) / Otro (respuestas observadas: CONUT, criterios GLIM)

44. ¿Se realiza una valoración rutinaria por parte del servicio de nutrición de todos los pacientes candidatos a cirugía colorrectal, al margen de su situación nutricional, con el objetivo de realizar una valoración de la ingesta nutricional y recibir consejo dietético en la fase preoperatoria?

No, para ningún paciente / No, solo aquellos con riesgo moderado o severo de desnutrición / No, solo pacientes oncológicos / No, solo pacientes con enfermedad inflamatoria intestinal / Sí, todos los pacientes quirúrgicos reciben sistemáticamente este tipo de valoración nutricional, independientemente de su situación nutricional / NS/NC

45. ¿Hacen un diagnóstico de desnutrición basado en criterios GLIM en los pacientes que se van a someter a cirugía colorrectal en su unidad?

No, para ningún paciente / No, solo aquellos con riesgo moderado o severo de desnutrición / No, solo pacientes oncológicos / No, solo pacientes con enfermedad inflamatoria intestinal / Sí, todos los pacientes quirúrgicos reciben sistemáticamente este tipo de valoración, independientemente de su situación nutricional / NS/NC

46. ¿Usan en su unidad herramientas para diagnóstico morfo-funcional de los pacientes que se van a someter a cirugía colorrectal de algún tipo?

No, para ningún paciente / No, solo aquellos con riesgo moderado o severo de desnutrición / No, solo pacientes oncológicos / No, solo pacientes con enfermedad inflamatoria intestinal / Sí, todos los pacientes quirúrgicos reciben sistemáticamente este tipo de valoración, independientemente de su situación nutricional / NS/NC

47. ¿Cuáles son las herramientas para diagnóstico morfofuncional que utilizan?

Dinamometría / Ecografía muscular / Impedanciometría bioeléctrica / TAC abdominal en L3 / NS/NC

48. En el paciente sin riesgo de desnutrición, ¿cómo de frecuente es que se le recomiende la realización de algún tipo de dieta específica o que reciba suplementación nutricional oral de algún tipo?

Nunca / Excepcional (<25%) / Raro (25–50%) / Frecuente (50–75%) / Rutinario (>75%) / Siempre

49. En caso de riesgo de desnutrición, ¿cuál de las siguientes representa mejor el abordaje habitual de suplementación nutricional oral?

Desde la propia consulta de cirugía se realiza suplementación nutricional oral en todos los casos con suplementos hiperproteicos / Desde la propia consulta de cirugía se realiza suplementación nutricional oral en todos los casos con inmunonutrientes / Se remite el paciente a la unidad de endocrinología y nutrición para una valoración clínica más completa, según su criterio / No hay una conducta fija; depende del tipo de paciente / No lo utilizamos

50. ¿Cuál es la duración de la suplementación nutricional oral en caso de prescribirse durante la fase preoperatoria?

Menos de 7 días / Entre 7 y 10 días / Todo el tiempo que transcurra desde la consulta preoperatoria hasta la intervención / Es variable dependiendo de las características del paciente y del resultado del test de cribado

51. ¿Existe en su hospital un protocolo concreto de manejo del paciente desnutrido o en riesgo severo de desnutrición en el que se establecen con claridad las distintas indicaciones de los tratamientos, controles posteriores y momento óptimo para la intervención?

Sí / No / NS/NC

52. En caso de producirse esa valoración para diagnóstico nutricional, ¿quién la realiza? *(multiple choice)*

Endocrinólogo / Nutricionista / Dietista / Equipo multidisciplinar / Otro (respuestas observadas: Cirujano, Enfermería, Internista)

53. Respecto al uso de suplementación nutricional, ¿cuál es la opción que mejor representa su rutina?

El uso es sistemático en todos los pacientes / El uso es en función de las herramientas de cribado, sin valoración nutricional posterior / Su uso es selectivo en función del diagnóstico tras la valoración nutricional específica / No existe una disciplina de equipo; depende de los gustos individuales del cirujano / Nunca se usan suplementos nutricionales orales de ningún tipo

54. Sobre los suplementos nutricionales orales hipercalóricos e hiperproteicos: *(multiple choice)*

Se prescriben en todos los pacientes que van a prehabilitación / Se prescriben sólo en los pacientes con cribado de desnutrición / Se prescriben sólo en pacientes con diagnóstico de desnutrición / Sólo se prescriben en la fase preoperatoria / Sólo se prescriben en la fase postoperatoria / Se prescriben en ambas fases

55. Sobre los suplementos nutricionales orales basados en inmunonutrición: *(multiple choice)*

Se prescriben en todos los pacientes que van a prehabilitación / Se prescriben sólo en los pacientes con cribado de desnutrición / Se prescriben sólo en pacientes con diagnóstico de desnutrición / Sólo se prescriben en la fase preoperatoria / Sólo se prescriben en la fase postoperatoria / Se prescriben en ambas fases

56. ¿Quién realiza la prescripción nutricional en los pacientes quirúrgicos?

Cirujano / Endocrino/nutricionista / Geriatra / Enfermera de práctica avanzada / Variable en función del tipo de paciente / NS/NC / Otro (respuesta observada: Anestesia y endocrino, Internista)

57. En caso de suplementación nutricional preoperatoria, ¿cuál de las siguientes se ajusta mejor a su diseño?

Desde el momento del diagnóstico se recomienda la suplementación nutricional oral / Se recomienda 10 días antes de la intervención / Se recomienda 7 días antes de la intervención / No existe una recomendación estándar; las decisiones se toman de forma individualizada

58. En cuanto a la suplementación nutricional oral postoperatoria, señale la que mejor representa su práctica clínica diaria:

Se inicia sistemáticamente de forma lo más inmediata posible / Se inicia de forma selectiva / Suele limitarse al ingreso / Suele durar al menos 10 días postoperatorios / La pauta de suplementación al alta suele durar semanas (especificar)

59. Si la pauta de suplementación suele durar semanas, especifique cuántas. *(open text)*

60. En cuanto a la valoración y recomendaciones dietéticas preoperatorias, ¿se recomienda algún tipo de dieta específica las semanas previas a la intervención? *(multiple choice)*

No, ninguna recomendación / Recomendación de evitar alimentos proinflamatorios / Recomendación de dieta rica en fibra / Recomendación de dieta pobre en residuos

61. ¿Han incorporado en su rutina de prehabilitación preoperatoria algún producto dirigido a modular la microbiota intestinal de cara a la intervención?

No / Sí

62. En caso afirmativo, especificar cuál. *(open text)*

Ejercicio físico

63. ¿Cuántas sesiones de entrenamiento semanal se prescriben?

Menos de 3 sesiones a la semana / Entre 3 y 5 sesiones a la semana / Más de 5 sesiones semanales / No existen pautas específicas

64. ¿Qué intensidad de ejercicio físico recomiendan dentro de su protocolo de prehabilitación?

Baja / Moderada / Alta / Lo desconozco

65. ¿En base a qué parámetro se establece dicha recomendación?

Frecuencia cardiaca / Unidades de VO2 máx / Lo desconozco / Otro (respuestas observadas: edad y comorbilidades, RPE, individualizado, protocolo IMPRICA)

66. ¿Qué tiempo de duración recomiendan para cada sesión?

Máximo 30 minutos / Entre 30 y 45 minutos / Entre 45 minutos y una hora / Más de una hora

67. ¿Quién prescribe las pautas de ejercicio en los pacientes quirúrgicos?

Cirujano / Geriatra / Rehabilitador / Fisioterapeuta / Otro (respuestas observadas: Anestesia, Enfermera de Procesos, Nutricionista, Preparador físico, EPA)

68. Señale las distintas formas de ejercicio físico que comprende su programa de prehabilitación en cirugía colorrectal: *(multiple choice)*

Estiramientos musculares y articulares / Equilibrio / Ejercicios de fuerza / Ejercicios de elasticidad / Fisioterapia respiratoria

69. ¿Qué tipo de régimen de ejercicios se prescriben?

Régimen hospitalario bajo supervisión siempre / Programa completamente ambulatorio sin supervisión de ningún tipo / Se pautan algunas sesiones supervisadas al principio y luego ambulatoriamente sin supervisión / Otro (respuestas observadas: recomendación de caminar en domicilio, bicicleta en hospital)

70. ¿Qué implica la pauta de ejercicios que se realiza?
Una valoración individualizada de cada paciente con recomendaciones específicas en cada caso / Pauta estándar basada en materiales gráficos que se entregan a los pacientes / No hay una pauta establecida / Depende de cuestiones individualizadas / NS/NC

71. ¿Qué tipo de entrenamiento utilizan?
LISS (ejercicio de larga duración y baja intensidad) / HIIT (intervalos de corta duración y alta intensidad) / NEAT (actividad no deportiva con gasto calórico extra, p. ej. subir escaleras) / No sabe/no contesta

72. ¿Qué supervisión realizan del progreso del paciente? *(multiple choice)*
Semanal / Quincenal / Mensual / Presencial / Telefónica / Telemática / Otro (respuestas observadas: ninguna supervisión, control por nutrición)

73. ¿Cómo se registran los entrenamientos de los pacientes?
Aplicaciones específicas de entrenamiento / Cuaderno o diario por parte del paciente / Tablas facilitadas previamente al paciente

74. ¿Utilizan alguna herramienta para medir la capacidad funcional de los pacientes o la actividad física de los mismos en su rutina habitual? *(multiple choice)*
Dispositivos de monitorización portátiles tipo wearables / Test Ejercicio Cardio-Pulmonar (CPET) / Test de los 6 minutos caminando / Cuestionario YPAS / Cuestionario CHAMPS / Test Time Up and Go / Valoración geriátrica integral / No se emplea ninguna

Manejo del estrés y la ansiedad

75. ¿Forma parte de su programa de prehabilitación el control del estrés y de la ansiedad emocional en el preoperatorio de los pacientes?
Sí / No

76. ¿Qué tipo de pacientes son remitidos a esta parte del circuito?
Todos sin distinción / Ninguno / Sólo los que lo solicitan / Sólo los que parecen deprimidos durante la entrevista / Sólo los que tienen una puntuación que sugiera riesgo de depresión y/o ansiedad en alguna escala validada

77. En caso de que su respuesta haya sido esta última, por favor indique cuál de las siguientes herramientas utiliza:
Escala de Yesavage / HADS / Ninguna / Otro (respuesta observada: HADS y SF-12)

78. Señale todos los recursos humanos de que dispone para la ejecución de esta parte de la prehabilitación: *(multiple choice)*
Psiquiatría / Psico-oncología / Enfermería con formación especializada / Enfermería sin formación especializada / Ninguna

79. ¿Qué tipo de estrategias, disciplina o ejercicios se utilizan en su unidad para evitar o combatir el posible estrés y ansiedad asociados al perioperatorio?
Terapia Mindfulness / Técnicas de relajación / Terapia cognitivo-conductual / Programación neurolingüística / Otro (respuestas observadas: derivación a psicooncología, ejercicios cognitivos)

80. En cuanto a las pautas que se dan para trabajar esta rama de la prehabilitación, señale la opción más adecuada:
Siempre son para practicarlas de forma individual ambulatoria / Siempre son para practicarlas de forma individual guiados por un terapeuta del tipo que sea / Siempre son para practicarlas en grupo guiados por un terapeuta / Existen ejercicios que se recomiendan de todos los tipos / La pauta que realizamos no se adecúa a ninguna de estas descripciones

Auditoría

81. Si la pauta que realizan no se adecúa a ninguna de las descripciones anteriores, por favor especifique: *(open text)*

82. ¿Mantienen en su centro una auditoría constante de todas las cuestiones que se han interrogado hasta ahora, más allá de la fase de implantación de una vía de cuidados para recuperación intensificada de los pacientes?
Sí / No / Solo de algunas cuestiones que siguen siendo potenciales áreas de mejora en nuestro centro / Algunas cuestiones sobre las que estamos desarrollando algún tipo de investigación

83. Si su respuesta ha sido alguna de las dos últimas, por favor especifique sobre qué materias. *(open text)*

Supplementary Materials Section S3. VAPPER Survey questionnaire — English version (83 items).

Respondent & Consent

1. Full name *(open text)*

2. Contact email address *(open text)*

3. Name of the hospital *(open text)*

Center Characteristics

4. Type of hospital

Public, non-university / Public, university / Private, non-university / Private, university

5. Hospital level of care

Tertiary / Secondary / District/community hospital

6. Is this an accredited colorectal surgery unit?

Basic unit / Advanced unit / Non-accredited unit / No such units exist

7. What position do you hold in your department?

Resident / Fellow in coloproctology / Attending physician with less than 10 years dedicated to coloproctology / Attending physician with more than 10 years dedicated to coloproctology / Head of Unit / Head of Department

Prehabilitation Program — General Structure

8. Is there an established preoperative prehabilitation program at your hospital?

Yes / No (skip to question 37)

9. If yes, is there a specific hospital clinical pathway so that any professional in the hospital is aware of it and it is correctly implemented?

Yes / No

10. Which of the following are part of the usual prehabilitation team? *(multiple choice)*

Surgeon / Anesthesiologist / Endocrinologist/nutritionist / Advanced practice nursing / Social worker / Physiotherapists / Rehabilitation physicians / Geriatricians / Psychiatrists/psychologists / Other

Target Population

11. Among the group participants, who coordinates patient activities during the prehabilitation period?

Case-management nurse / Advanced practice nurse / Anesthesia / Surgery / Geriatrics / Other

12. Is there any exclusion criterion for the application of prehabilitation-type programs at your center? *(multiple choice)*

Obese patients / Diabetic patients / ASA IV patients / Young patients (<70 years) / Non-frail patients / Patients with metastatic disease / Non-oncological patients / We do not consider any exclusion criterion

13. Among elderly patients (over 70 years old), is frailty screening systematically performed to select candidates for prehabilitation?

No / G8 / Fried Frailty Scale / Timed Up and Go test / Edmonton Frail Scale / Short Physical Performance Battery / Clinical Frailty Scale (CFS) / Other

14. In the case of a positive screening, is a consultation performed for a precise frailty diagnosis using Comprehensive Geriatric Assessment (CGA)?

No / Yes / Selectively

15. If the answer is "selectively," please specify in which cases it is performed. *(open text)*

16. Who provides care in that frailty-diagnosis consultation?

Geriatrics / Anesthesia / Nursing staff with dedicated training for this purpose / Unknown / Other

Preoperative Education

17. Regarding the information given to patients at the preoperative visit, is there an additional consultation within the pathway specifically to discuss perioperative care, including prehabilitation and enhanced recovery, or must everything be covered in a single visit?

Single consultation / There is an additional consultation separate from the surgical visit / Don't know / no answer

18. If an additional consultation exists as a resource to inform patients of all these aspects, who coordinates that consultation?

Anesthesia / Advanced practice nursing / Patient education school / Geriatrics / Other

19. Has your center developed any type of document or infographic to support the information given to patients preoperatively?

Nothing has been developed at our center; verbal information only / An informal document exists / Detailed graphic material has been developed / Online material has been developed

Preoperative Optimization

20. Are surgical risk calculators applied to estimate the expected morbidity/mortality for each specific case?

No, we never use anything beyond the anesthesiologists' ASA classification / Only in specific cases with a frail appearance / Only in specific cases with a frailty diagnosis / Used only occasionally, without standardized criteria regarding patient profile or type of intervention / We apply them for scientific/academic interest, but not clinically / Always

21. If applicable, please indicate which of the following tools you use: *(multiple choice)*

P-POSSUM / CR-POSSUM / ACS NSQIP (ACS Surgical Risk Calculator, riskcalculator.facs.org) / E-PASS / DGAV / Corrected Cardiac Risk Index (Lee Index) / Other

22. Is smoking status assessed and, if applicable, are specific cessation recommendations given?

Recorded as medical history only / Cessation is recommended / A dedicated infographic exists to facilitate smoking cessation / Patient is referred to a specialized unit / Assessing smoking status is not part of routine practice

23. Is alcohol use assessed and, if applicable, are specific cessation recommendations given?

Recorded as medical history only, nothing further / Cessation is actively recommended / A dedicated infographic exists to facilitate alcohol cessation / Patient is referred to a specialized unit / Assessing alcohol use is not part of routine practice

Outreach Sessions

24. In the last 5 years, have outreach sessions been held to publicize the goals and potential results of these programs?

Yes / No

25. If yes, please select all audiences these outreach sessions targeted: *(multiple choice)*

Patients / Healthcare staff: nursing and nursing assistants / Healthcare staff: physiotherapists / Healthcare staff: surgical department physicians / Healthcare staff: physicians from other non-surgical specialties involved (e.g., anesthesia, rehabilitation, endocrinology) / Healthcare staff: primary care / Hospital management/administration

Duration

26. What is the duration of the prehabilitation program?

Two weeks / Three weeks / Four weeks / A minimum of four weeks, sometimes extended depending on the patient / Depending on patient profile: four weeks for frail patients and two weeks for non-frail patients

Advanced Practice Nursing (APN)

27. Do you have advanced practice nursing (APN) for continuous care of patients included in a prehabilitation program?

Yes / No

28. If APN is available for your prehabilitation pathway, select the option that best matches your reality: *(multiple choice)*

Performs their own preoperative assessment / Conducts in-person follow-up of the process / Conducts telephone follow-up of the preoperative process / Conducts follow-up after hospital discharge / Their role is purely administrative; more of a case manager than true APN

Quality of Life (QoL)

29. Do you use any scale for perioperative quality-of-life assessment?

No / Yes / Only in prehabilitation patients who are candidates for a study requiring it / Don't know/no answer

30. If yes, which scale is used? *(open text)*

PROMs & PROMIS

31. Do you use any scale for PROM/PROMIS assessment?

No / Yes / Only in prehabilitation patients who are candidates for a study requiring it / Don't know/no answer

32. If yes, which scale is used? *(open text)*

Patient School / Group Sessions

33. Are patient schools or patient group meetings a tool routinely used at your center?

Yes / No

34. If yes, indicate all disciplines that have developed workshops or activities of this type: *(multiple choice)*

Anesthesia / Nutrition / Psychiatry/Psychology / Generic ERAS-type perioperative pathway / Rehabilitation / Geriatrics / None / Unknown

Use of New Technologies

35. Does your unit rely in any way on new technologies (digital tools, apps, wearables) to apply prehabilitation principles?

Yes, we recommend it in all cases / We have tools that we use in selected cases / We have tried it, but abandoned it / We do not have it and have never tried it

36. If you have never worked in this area, would you be willing to try it if a platform existed that had proven satisfactory for patients?

Yes, we would be willing to try it / I don't think it would work for our patients / I don't think such tools exist

Preoperative Anemia Management

37. Is preoperative anemia screening a routine practice for patients entering the enhanced recovery clinical pathway?

Always / Only oncological patients / There is not enough time between the pre-anesthesia assessment and scheduling of the intervention, so this screening is not performed / Not part of routine practice; only in cases of severe anemia or where anemia is the main "symptom"

38. When anemia is diagnosed, its management is carried out:

Within the conventional pre-anesthesia consultation / There is a specific "blood-saving" consultation for treating preoperative anemia / By the Hematology department / By the Internal Medicine department / The Surgery team itself manages preoperative anemia / Other

39. Which of the following therapeutic alternatives are part of your usual arsenal for treating preoperative anemia? *(multiple choice)*

Oral iron / Intravenous iron / Erythropoietin administration / Administration of other erythropoiesis-stimulating agents / Don't know/no answer

40. Do you consider that your unit genuinely applies restrictive measures for red blood cell transfusion?

Yes / No / I have no opinion on this

41. Does your hospital have a specific protocol for managing patients with preoperative anemia that clearly establishes treatment indications, follow-up controls, etc.?

Yes / No / Unknown

Nutrition

42. During nutritional assessment, is the patient's weight and height measured?

Always / Never / Depending on responses regarding weight loss / Don't know/no answer

43. Do you perform any type of malnutrition screening for patients included in the prehabilitation program? *(multiple choice)*

No / Mini Nutritional Assessment (MNA) / Malnutrition Universal Screening Tool (MUST) / Subjective Global Assessment (SGA) / Nutritional Risk Screening 2002 (NRS 2002) / Simplified Nutritional Assessment Questionnaire (SNAQ) / Perioperative Nutrition Screen (PONS) / Other

44. Is a routine assessment performed by the nutrition department for all colorectal surgery candidates, regardless of nutritional status, to assess dietary intake and provide dietary advice preoperatively?

No, for no patients / No, only those with moderate or severe risk of malnutrition / No, only oncological patients / No, only patients with inflammatory bowel disease / Yes, all surgical patients systematically receive this type of nutritional assessment, regardless of their nutritional status / Don't know/no answer

45. Do you make a malnutrition diagnosis based on GLIM criteria in patients who will undergo colorectal surgery in your unit?

No, for no patients / No, only those with moderate or severe risk of malnutrition / No, only oncological patients / No, only patients with inflammatory bowel disease / Yes, all surgical patients systematically receive this type of assessment, regardless of their nutritional status / Don't know/no answer

46. Do you use any tools for morphofunctional diagnosis in patients who will undergo colorectal surgery?

No, for no patients / No, only those with moderate or severe risk of malnutrition / No, only oncological patients / No, only patients with inflammatory bowel disease / Yes, all surgical patients systematically receive this type of assessment, regardless of their nutritional status / Don't know/no answer

47. Which morphofunctional diagnostic tools do you use?

Dynamometry / Muscle ultrasound / Bioelectrical impedance analysis / Abdominal CT at L3 / Don't know/no answer

48. In patients without risk of malnutrition, how often is a specific diet or some form of oral nutritional supplementation recommended?

Never / Exceptional (<25%) / Rare (25–50%) / Frequent (50–75%) / Routine (>75%) / Always

49. In patients with risk of malnutrition, which of the following best represents the usual approach to oral nutritional supplementation?

Oral nutritional supplementation with high-protein supplements is provided in all cases directly from the surgical clinic / Oral nutritional supplementation with immunonutrients is provided in all cases directly from the surgical clinic / The patient is referred to the endocrinology/nutrition unit for a more complete clinical assessment, at their discretion / There is no fixed approach; it depends on the type of patient / We do not use it

50. What is the duration of oral nutritional supplementation when prescribed during the preoperative phase, in case of risk of malnutrition?

Less than 7 days / Between 7 and 10 days / The entire time between the preoperative visit and the intervention / Variable, depending on patient characteristics and screening test results

51. Does your hospital have a specific protocol for managing malnourished patients or those at severe risk of malnutrition, clearly establishing treatment indications, follow-up controls, and optimal timing for surgery?

Yes / No / Don't know/no answer

52. When this nutritional-diagnosis assessment takes place, who performs it? *(multiple choice)*

Endocrinologist / Nutritionist / Dietitian / Multidisciplinary team / Other

53. Regarding the use of nutritional supplementation, which option best represents your routine?

Use is systematic in all patients / Use is based on screening tools, without any subsequent nutritional assessment / Use is selective, based on the diagnosis following a specific nutritional assessment / There is no team-wide practice; it depends on the surgeon's individual preference / Oral nutritional supplements of any kind are never used

54. Regarding hypercaloric/high-protein oral nutritional supplements: *(multiple choice)*

Prescribed for all patients undergoing prehabilitation / Prescribed only for patients with a positive malnutrition screening / Prescribed only for patients with a malnutrition diagnosis / Prescribed only in the preoperative phase / Prescribed only in the postoperative phase / Prescribed in both phases

55. Regarding immunonutrition-based oral nutritional supplements: *(multiple choice)*

Prescribed for all patients undergoing prehabilitation / Prescribed only for patients with a positive malnutrition screening / Prescribed only for patients with a malnutrition diagnosis / Prescribed only in the preoperative phase / Prescribed only in the postoperative phase / Prescribed in both phases

56. Who prescribes nutrition in surgical patients?

Surgeon / Endocrinologist/nutritionist / Geriatrician / Advanced practice nurse / Variable, depending on patient type / Don't know/no answer / Other

57. In the case of preoperative nutritional supplementation, which of the following best fits your design?

Oral nutritional supplementation is recommended from the moment of diagnosis / Recommended 10 days before the intervention / Recommended 7 days before the intervention / There is no standard recommendation; decisions are made on an individual basis

58. Regarding postoperative oral nutritional supplementation, indicate the option that best represents your daily clinical practice:

Started systematically as soon as possible / Started selectively / Usually limited to the inpatient stay / Usually lasts at least 10 postoperative days / The postoperative supplementation regimen usually lasts several weeks (please specify)

59. If the supplementation regimen usually lasts several weeks, please specify how many. *(open text)*

60. Regarding preoperative dietary assessment and recommendations, is any specific type of diet recommended in the weeks prior to the intervention? *(multiple choice)*

No, no recommendation / Recommendation to avoid pro-inflammatory foods / Recommendation of a high-fiber diet / Recommendation of a low-residue diet

61. Have you incorporated any product aimed at modulating the gut microbiota ahead of surgery into your preoperative prehabilitation routine?
No / Yes

62. If yes, please specify which one. *(open text)*

Exercise

63. How many weekly training sessions are prescribed?
Fewer than 3 sessions per week / Between 3 and 5 sessions per week / More than 5 sessions per week / There are no specific guidelines

64. What exercise intensity do you recommend within your prehabilitation protocol?
Low / Moderate / High / Unknown

65. On what parameter is this recommendation based?
Heart rate / VO2 max units / Unknown / Other

66. What session duration do you recommend?
Maximum 30 minutes / Between 30 and 45 minutes / Between 45 minutes and one hour / More than one hour

67. Who prescribes the exercise regimen for surgical patients?
Surgeon / Geriatrician / Rehabilitation physician / Physiotherapist / Other

68. Please indicate the different forms of physical exercise included in your colorectal-surgery prehabilitation program: *(multiple choice)*
Muscle and joint stretching / Balance training / Strength exercises / Flexibility exercises / Respiratory physiotherapy

69. What type of exercise regimen is prescribed?
Always fully supervised in a hospital setting / Completely outpatient program with no supervision of any kind / Some supervised sessions are scheduled at the start, followed later by an outpatient regimen / Other

70. What does the exercise regimen involve?
An individualized assessment of each patient with specific recommendations for each case / A standard regimen based on printed materials handed to patients / There is no established regimen / It depends on individual circumstances / Don't know/no answer

71. What type of training do you use?
LISS (low intensity steady state) / HIIT (high intensity interval training) / NEAT (non-exercise activity thermogenesis) / Don't know/no answer

72. What supervision of patient progress is carried out? *(multiple choice)*
Weekly / Biweekly / Monthly / In-person / By phone / Remote/telematic / Other

73. How are patients' training sessions recorded?
Specific training apps / Patient logbook/diary / Charts previously provided to the patient

74. Do you use any tool to measure patients' functional capacity or physical activity in your routine practice? *(multiple choice)*
Wearable monitoring devices / Cardiopulmonary Exercise Testing (CPET) / 6-minute walk test / YPAS questionnaire / CHAMPS questionnaire / Timed Up and Go test / Comprehensive geriatric assessment / None used

Stress and Anxiety Management

75. Does your prehabilitation program include managing preoperative emotional stress and anxiety in patients?
Yes / No

76. What type of patients are referred to this part of the pathway?
All, without distinction / None / Only those who request it / Only those who appear depressed during the interview / Only those with a score suggesting risk of depression and/or anxiety on a validated scale

77. If your answer was the last option, please indicate which of the following tools you use:
Yesavage Scale / HADS / None / Other

78. Please indicate all human resources available for carrying out this part of prehabilitation: *(multiple choice)*
Psychiatry / Psycho-oncology / Nurses with specialized training / Nurses without specialized training / None

79. What strategies, disciplines, or exercises are used in your unit to prevent or manage perioperative stress and anxiety?

Mindfulness therapy / Relaxation techniques / Cognitive-behavioral therapy / Neuro-linguistic programming / Other

80. Regarding the guidance given for this branch of prehabilitation, indicate the most appropriate option:

Always meant to be practiced individually, on an outpatient basis / Always meant to be practiced individually, guided by a therapist of any kind / Always meant to be practiced in a group, guided by a therapist / There are exercises recommended of all these types / The approach we follow does not match any of these descriptions

Audit

81. If the approach you follow does not match any of the above descriptions, please specify: *(open text)*

82. Do you maintain ongoing auditing of all the issues addressed so far at your center, beyond the implementation phase of the enhanced recovery clinical pathway?

Yes / No / Only for some issues that remain potential areas of improvement at our center / Some issues on which we are currently conducting some type of research

83. If your answer was one of the last two options, please specify on which topics. *(open text)*
